# A rare type of polyp in the duodenum

**DOI:** 10.1002/ccr3.7969

**Published:** 2023-10-04

**Authors:** Kumi Itami, Takaaki Yoshikawa, Shujiro Yazumi

**Affiliations:** ^1^ Department of Gastroenterology and Hepatology Medical Research Institute Kitano Hospital Osaka Japan

**Keywords:** duodenum, endoscopic resection, Peutz–Jeghers syndrome, polyp

## Abstract

Although solitary P‐J type hamartomatous polyp in the duodenum is rare, the polyp has malignant potential. We should recognize the entity and resect it with a safety margin in case the polyp exhibits an irregular form.

## Graphical abstract

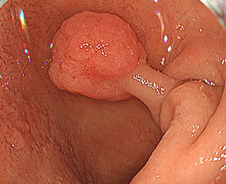

## 1 INTRODUCTION

**FIGURE 1:**
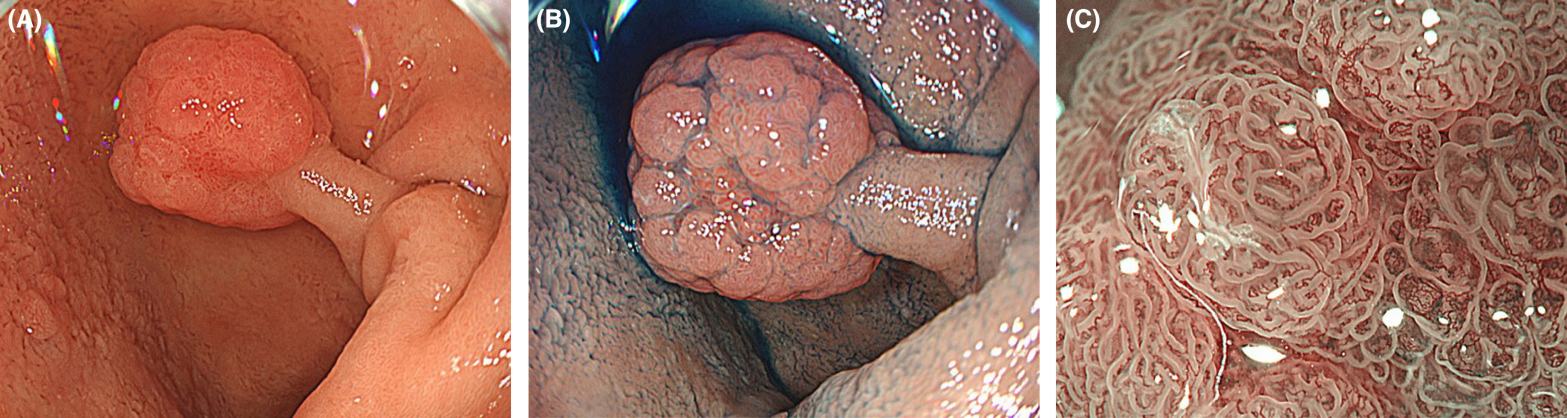
A: Duodenal polyp measuring 15 mm in diameter were located in the superior duodenal angle. B: Indigocarmine dye contrast imaging clarified the demarcation line of the polyp. C: Narrow band imaging with magnification showed villi morphology and regular vessel pattern.

**FIGURE 2:**
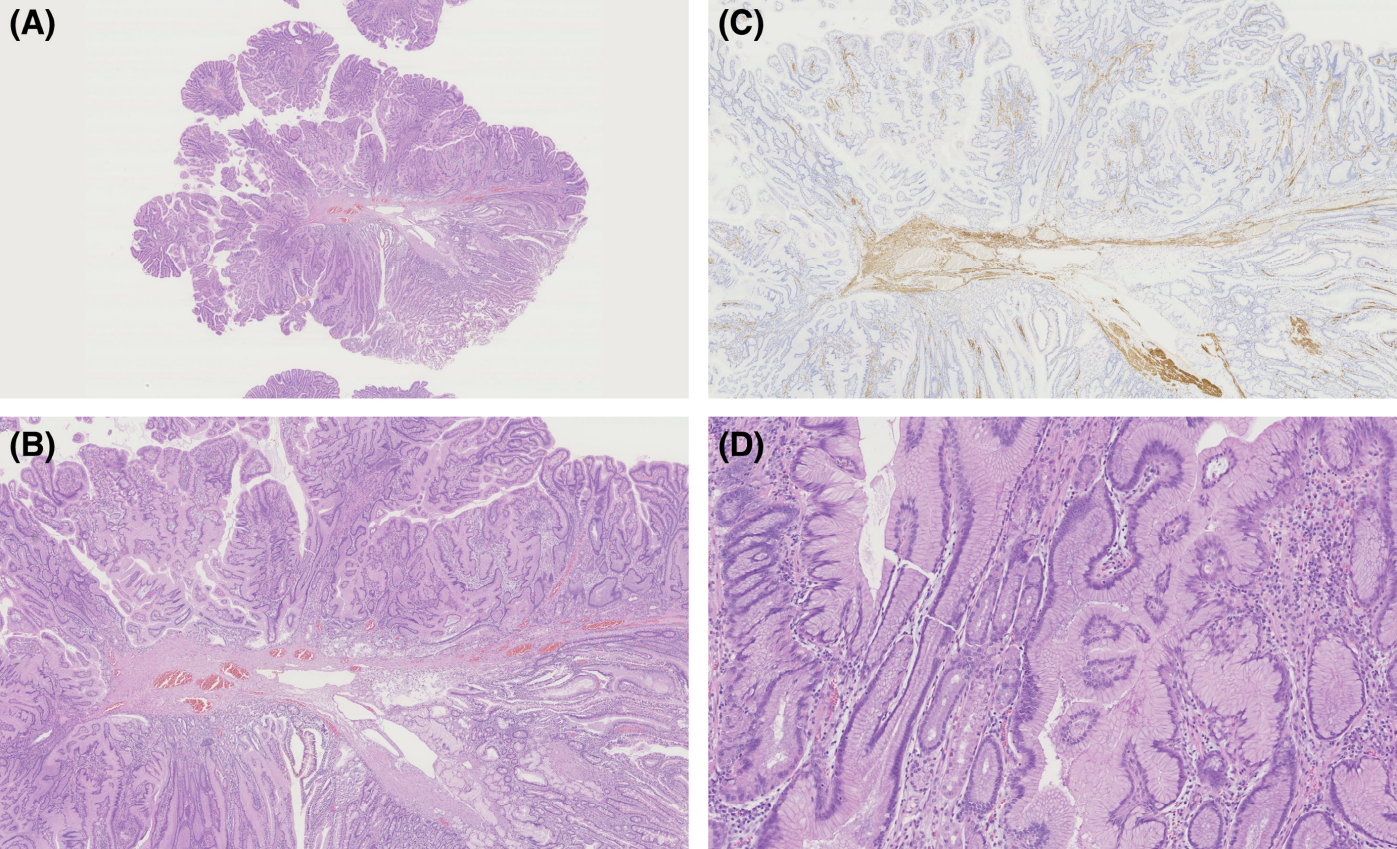
(A and B): Histological specimen showed branching bundles of smooth muscle fibers covered by hyperplastic duodenal mucosa. (Hematoxylin and eosin staining, ×0.7 and ×2, respectively). (C): Immunostaining for desmin clarified branching bundles of smooth muscle fibers. (×2). (D): Histological specimen did not show any evidence of dysplasia. (×10).

A 61‐year‐old woman was referred to our department for screening of upper gastrointestinal tract. She did not have any medical history of malignancy nor any specific family history, including Peutz–Jeghers (P‐J) syndrome. Physical examination showed normal, and there was no pigmentation in the skin and the lip. We performed esophagogastroduodenoscopy, and it revealed a single 0‐I polyp with 15 mm diameter located on the lateral wall of the superior duodenal angle (Figure 1A–C). The polyp showed irregularly lobular surface with slight redness with a normal light observation (Figure 1A). Indigocarmine spraying clarified border of the polyp (Figure 1B). Narrow band imaging revealed villi morphology but could not detect irregular vessel pattern (Figure 1C). Endoscopic mucosal resection was performed to obtain a whole sample. Histopathological examination showed branching bundles of smooth muscle fibers covered by hyperplastic duodenal mucosa with no dysplasia (Figure 2A–D). What is the diagnosis of the polyp?

Since branching bundles of smooth muscle fibers and hyperplastic mucosa were distinctive characters of P‐J type polyp, the polyp was diagnosed as a P‐J type hamartomatous polyp. In addition, the patient did not have family history and all the other clinical features of P‐J syndrome, the polyp was finally diagnosed as a solitary P‐J type hamartomatous polyp. The polyp did not harbor the malignant component and was resected completely.

## 2 DISCUSSION

P‐J syndrome is a rare, autosomal‐dominant disorder defined by the development of characteristic polyps throughout the gastrointestinal tract and mucocutaneous pigmentation. By contrast, P‐J type polyp whose patient does not have characteristic symptoms or family history of P‐J was described as a solitary P‐J type hamartomatous polyp. Solitary P‐J type hamartomatous polyps are commonly seen in the large intestine, however, rare in duodenum.^1^ Although there is no malignant change in our case, a previous report showed that 14.8% of P‐J type hamartomatous polyps harbored adenocarcinoma component.^2^ Although solitary P‐J type hamartomatous polyp is rare, we should recognize the entity and resect it with safety margin because of its malignant potential.

## AUTHOR CONTRIBUTIONS

**Kumi Itami:** Conceptualization; data curation; investigation; methodology; resources; writing – original draft. **Takaaki Yoshikawa:** Conceptualization; methodology; supervision; writing – review and editing. **Shujiro Yazumi:** Conceptualization; project administration; supervision; writing – review and editing.

## CONFLICT OF INTEREST STATEMENT

No potential conflict of interest relevant to this article was reported.

## CONSENT

Written informed consent was obtained from the patient to publish this report in accordance with the journal's patient consent policy.

## Data Availability

The data that support the findings of this study are available from the corresponding author upon reasonable request.
